# A Role for SKN-1/Nrf in Pathogen Resistance and Immunosenescence in *Caenorhabditis elegans*

**DOI:** 10.1371/journal.ppat.1002673

**Published:** 2012-04-26

**Authors:** Diána Papp, Péter Csermely, Csaba Sőti

**Affiliations:** Department of Medical Chemistry, Semmelweis University, Budapest, Hungary; Massachusetts General Hospital, Harvard Medical School, United States of America

## Abstract

A proper immune response ensures survival in a hostile environment and promotes longevity. Recent evidence indicates that innate immunity, beyond antimicrobial effectors, also relies on host-defensive mechanisms. The *Caenorhabditis elegans* transcription factor SKN-1 regulates xenobiotic and oxidative stress responses and contributes to longevity, however, its role in immune defense is unknown. Here we show that SKN-1 is required for *C. elegans* pathogen resistance against both Gram-negative *Pseudomonas aeruginosa* and Gram-positive *Enterococcus faecalis* bacteria. Exposure to *P. aeruginosa* leads to SKN-1 accumulation in intestinal nuclei and transcriptional activation of two SKN-1 target genes, *gcs-1* and *gst-4*. Both the Toll/IL-1 Receptor domain protein TIR-1 and the p38 MAPK PMK-1 are required for SKN-1 activation by PA14 exposure. We demonstrate an early onset of immunosenescence with a concomitant age-dependent decline in SKN-1-dependent target gene activation, and a requirement of SKN-1 to enhance pathogen resistance in response to longevity-promoting interventions, such as reduced insulin/IGF-like signaling and preconditioning H_2_O_2_ treatment. Finally, we find that *wdr-23(RNAi)*-mediated constitutive SKN-1 activation results in excessive transcription of target genes, confers oxidative stress tolerance, but impairs pathogen resistance. Our findings identify SKN-1 as a novel regulator of innate immunity, suggests its involvement in immunosenescence and provide an important crosstalk between pathogenic stress signaling and the xenobiotic/oxidative stress response.

## Author Summary

Innate immunity promotes survival by combating pathogenic threat. During infection, tissue damage is induced both by invading pathogens and immune effectors such as toxins and free radicals. Therefore, it is important to elucidate by what self-protective mechanisms the host defends itself against pathogenic stress. The conserved SKN-1 protein of the roundworm *Caenorhabditis elegans* directs a detoxification response neutralizing harmful compounds as well as confers tolerance to oxidative stress. Here we identify SKN-1 as a novel regulator of *C. elegans* innate immunity. We show that SKN-1 contributes to resistance against infection caused by two bacterial pathogens. Components of a pathogen-responsive signaling pathway are required to activate SKN-1 in intestinal cells at the site of infection. Moreover, the SKN-1-dependent response to pathogen exposure declines during aging, whereas mild metabolic and oxidative stresses, known to extend lifespan, evoke a SKN-1-dependent boosting of immunity. Finally, we find that elimination of an inhibitory protein leads to an excessive activation of SKN-1 and impairs pathogen resistance. Thus, SKN-1 integrates various chemical, metabolic and microbial signals to elicit a self-protective detoxification response, which promotes innate immunity and may be relevant to diseases and aging of the human immune system.

## Introduction

A proper immune response ensures survival in a hostile environment and contributes to longevity. The nematode *Caenorhabditis elegans* provides a valuable genetic tool for studying innate immunity and various aspects of host-pathogen interactions. During infection, both bacterial virulence factors and host antimicrobial defense mechanisms present oxidative and proteotoxic noxae inducing tissue-damage, especially in the intestine [1]–[5]. Accordingly, several self-protective stress-response regulators including the forkhead transcription factor DAF-16/FOXO [6], the heat shock transcription factor HSF-1 [7] and the X-box binding protein 1 (XBP-1) [8] are required for robust immunity. Moreover, the DAF-16-regulated antioxidant enzymes SOD-3 and CTL-2 contribute to immunity by protecting intestinal cells from reactive oxygen species during exposure to *Enterococcus faecalis* [9]. Strikingly, hyper-activation of DAF-16 enhances susceptibility to bacterial infection [10].

These data illustrate a critical role of stress response in innate immunity, and raise questions about the co-ordination of antimicrobial and host-defense mechanisms. Antimicrobial responses are mediated by a canonical p38 mitogen-activated protein kinase (MAPK) pathway, which is conserved from nematodes to humans [11], [12]. Besides, the insulin/IGF-like signaling (IIS) and TGF-β pathways are also involved in the regulation of the pathogen-specific immune response in *C. elegans* [13], [14]. Both p38 MAPK and IIS pathways regulate the Nrf1/2/3 ortholog SKN-1, a transcription factor that orchestrates both oxidative and xenobiotic stress responses in *C. elegans* [15], [16]. However, the involvement of SKN-1 in the regulation of pathogen stress response is unknown.

In nematodes, three SKN-1 isoforms exist. While the function of SKN-1A has not been elucidated yet, SKN-1B and C provide distinct biological functions. SKN-1B is expressed in the ASI neurons, and mediates lifespan extension in response to dietary restriction [17]. In contrast, intestinal SKN-1C is required for oxidative stress resistance and contributes to longevity by reduced IIS [15]. SKN-1 activity is regulated by phosphorylation and degradation. Under normal conditions, inhibitory phosphorylations by GSK-3 and IIS kinases, AKT-1/2 and SGK-1, retain SKN-1 in the cytosol [15], [18], where it is rapidly targeted to proteasomal degradation by the WD40 repeat protein WDR-23 [19], [20]. In response to oxidative stress, the p38 MAPK ortholog PMK-1 phosphorylates SKN-1, which then translocates to the nuclei of intestinal cells and induces transcription of phase 2 detoxification genes [16].

Here we report that SKN-1 is required for pathogen resistance against both Gram-negative *P. aeruginosa* and Gram-positive *E. faecalis* bacteria, consistently with an independent study [21] published after submission of this paper. We further demonstrate a Toll and Interleukin-1 Receptor domain protein (TIR-1)/PMK-1-dependent SKN-1 activation upon *P. aeruginosa* infection. Moreover, we show a gradual decrease of pathogen resistance and of the activation of SKN-1-dependent targets during aging, and a requirement of SKN-1 to boost immunity in response to longevity-promoting manipulations, such as reduced IIS and preconditioning H_2_O_2_ treatment. Finally, we find that hyper-activation of SKN-1 impairs pathogen resistance. Our results indicate an intricate regulation of innate immunity by SKN-1 and links pathogenic stress signaling to the xenobiotic stress response.

## Results

### SKN-1 is required for bacterial pathogen resistance in *C. elegans*

**Figure 1:**
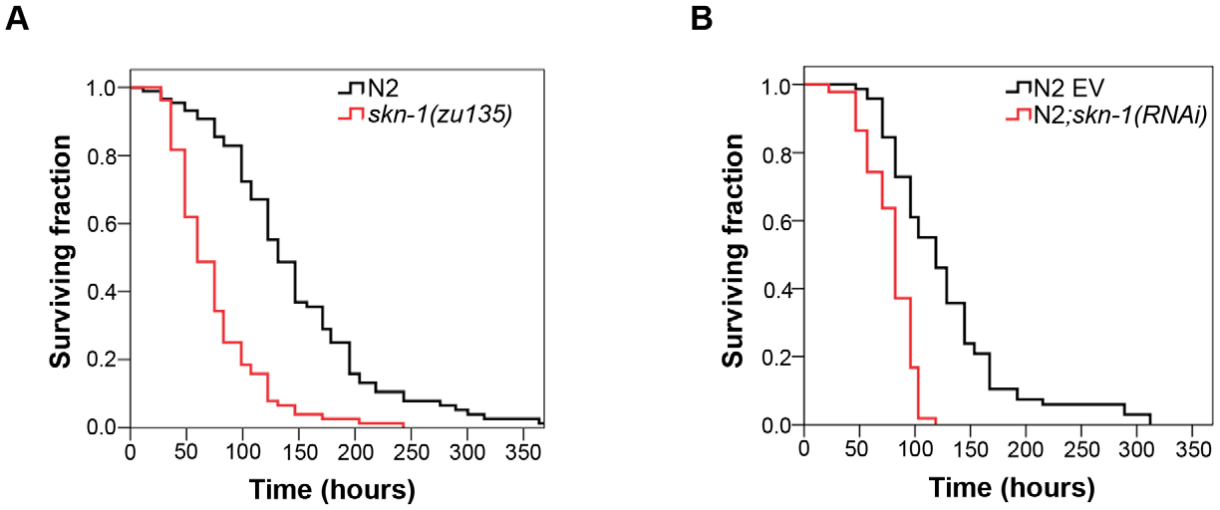
SKN-1 is required for bacterial pathogen resistance. (**A, B**) Increased susceptibility to *Pseudomonas aeruginosa* PA14 occurs in both *skn-1(zu135)* mutant (p<0.0001) and *skn-1(RNAi)* nematodes (p<0.0001). Killing assays were performed with at least 90 young adult animals in each condition. EV: empty vector RNAi.

To study the role of SKN-1 in *C. elegans* immunity, we examined the pathogen resistance of animals in the absence of SKN-1. *skn-1(zu135)* allele is considered to be a genetic null mutation as it creates a premature stop codon that affects all SKN-1 isoforms [15]. *skn-1(zu135)* mutant worms were first exposed to the Gram-negative *Pseudomonas aeruginosa* (PA14) strain. As *skn-1(zu135)* mutants are sterile, we eliminated the difference between them and wild-type N2 strain arising from the ‘bag of worms’ phenotype, a major contributor to killing. To this end, germline development was inhibited by silencing *cdc-25.1*, required for embryonic mitosis and meiosis. *cdc-25.1(RNAi)* animals exhibit extended survival on pathogenic bacteria, as reported previously [21], [22]. In these conditions, we observed an increased susceptibility of *skn-1(zu135)* mutants to PA14 (Figures 1A and S1A, Tables S1A and S1E). This result was confirmed by using *skn-1(RNAi)* (Figures 1B and S1B, Table S1A and S1E). Furthermore, when animals were exposed to the Gram-positive *Enterococcus faecalis* SdB262 strain, both *skn-1(zu135)* and *skn-1(RNAi)* exhibited significantly decreased survival, though in this case the absence of SKN-1 exerted a more modest effect (Figures S1C and S1D, Table S1E). These results suggest a requirement of SKN-1 for the efficient immune response against two distinct bacterial pathogens. In subsequent experiments, we focused on further defining the role of SKN-1 in the antibacterial response against *P. aeruginosa*.

### *P. aeruginosa* infection triggers SKN-1 activation

**Figure 2:**
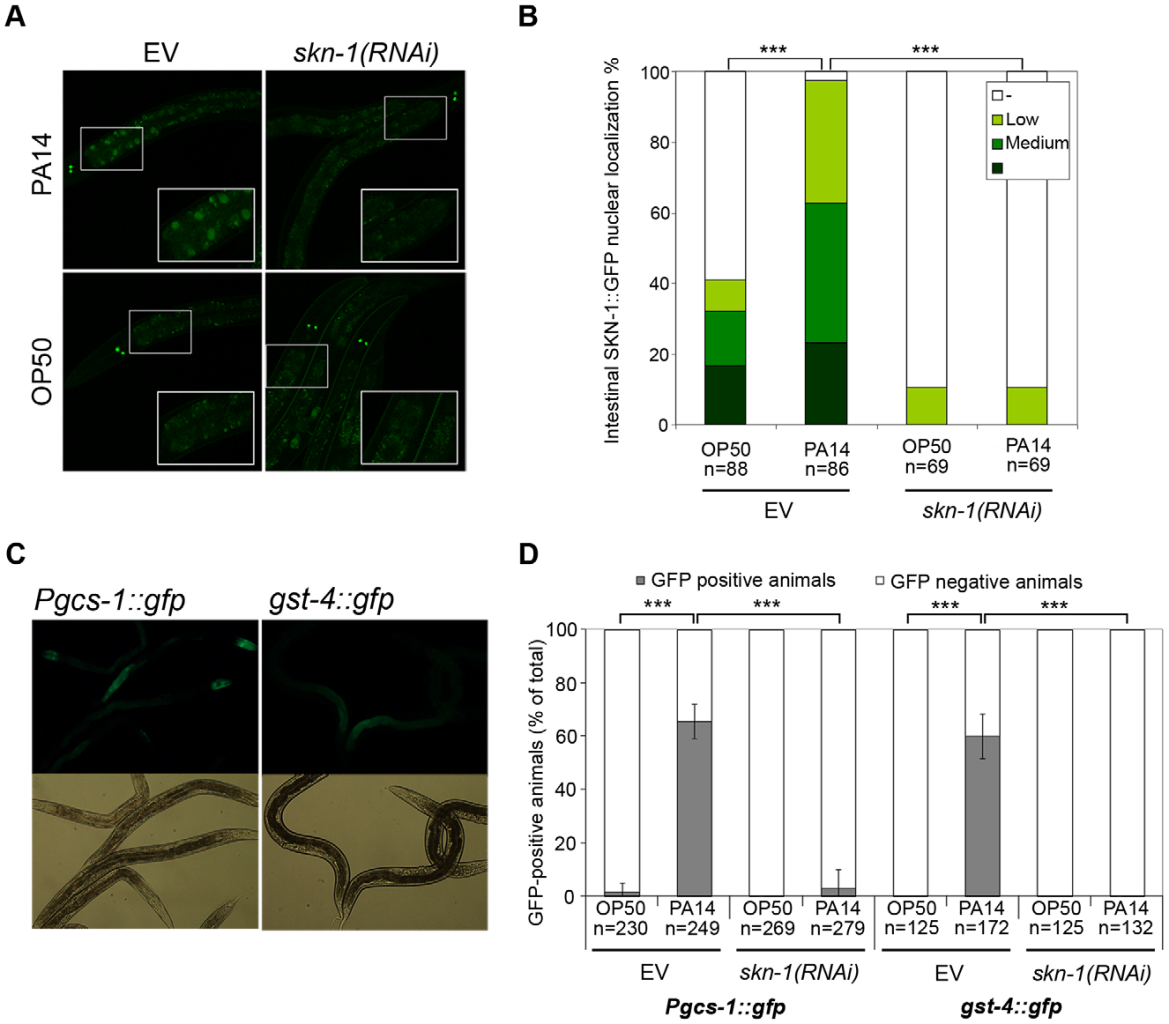
*P. aeruginosa* infection activates SKN-1. (**A**) Representative epifluorescence image demonstrating the translocation of SKN-1::GFP in the *Is007*[SKN-1::GFP] strain to intestinal nuclei in L3 larvae, fed by the empty vector or *skn-1* dsRNA, upon a 5-hour exposure to *P. aeruginosa* PA14. Note that the intestinal tissue displays autofluorescence, and in the ASI neurons SKN-1::GFP is not silenced by *skn-1* RNAi treatment. (**B**) Quantification of SKN-1 nuclear translocation from data shown on panel (A). SKN-1::GFP-positive nuclei were counted in the intestine of 78 animals. “Low” refers to animals in which SKN-1::GFP was detected in less than 5 intestinal nuclei, while “high” indicates that SKN-1::GFP signal was present in more than 15 intestinal nuclei. (**C**) Representative epifluorescence microscopic image showing intestinal expression of *Pgcs-1*::GFP and GST-4::GFP in L3 larvae upon a 24-hour PA14 exposure. Images of control animals incubated on OP50 bacteria are shown in Figure S2. (**D**) Quantification of reporter expression demonstrating the SKN-1-dependence of the response. Data were obtained from panel (C) completed with the data of *skn-1(RNAi)* animals. Microscopic images are representatives of 3 independent experiments. EV: empty vector RNAi.

To investigate if SKN-1 nuclear translocation occurs upon PA14 exposure, we incubated *skn-1::gfp* L3 larvae on *P. aeruginosa* lawn for 5 hours. We found a massive accumulation of SKN-1::GFP in intestinal nuclei of infected larvae, compared to control animals fed by the non-pathogenic OP50 *Escherichia coli* strain (Figures 2A and 2B). The specificity of this response was demonstrated by a complete inhibition using a *skn-1*-specific double-stranded RNA. To reveal a SKN-1-dependent transcriptional activation upon PA14 infection, we examined the *Pgcs-1::gfp* and g*st-4::gfp* reporter strains. While *gcs-1* is regulated exclusively by SKN-1, *gst-4* is under the mutual control of both DAF-16 and SKN-1 [15]. We observed an effective intestinal induction of fluorescence to comparable extent in both strains in response to a 24 h-exposure of PA14 (Figures 2C and 2D). Both the *gcs-1* promoter activation and the GST-4 expression were significantly suppressed by feeding worms with *skn-1(RNAi)*, indicating the specific requirement of SKN-1 to elicit these responses. Thus, PA14 infection induces nuclear translocation of SKN-1 and transcriptional activation of its targets.

### The TIR-1/PMK-1 pathway controls SKN-1 activation upon *P. aeruginosa* infection

**Figure 3:**
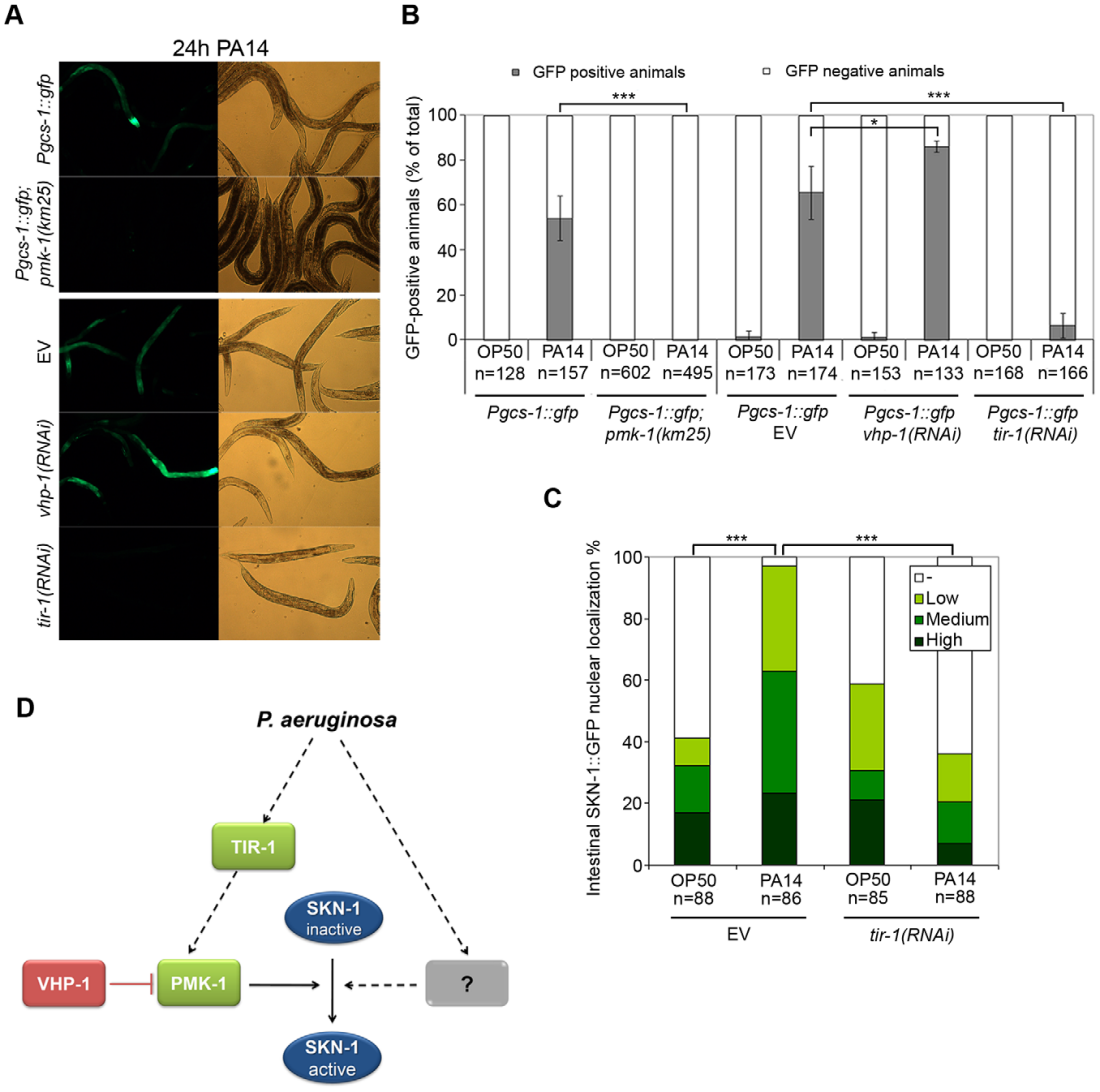
The pathogen response-specific TIR-1 and p38 MAPK PMK-1 are required for SKN-1 activation upon *P. aeruginosa* infection. (**A**) Representative epifluorescence microscopic images showing the expression of *Pgcs-1*::GFP in *pmk-1(km25)* mutants as well as in the p38 MAPK phosphatase *vhp-1(RNAi)*, and the Toll/IL-1 resistance (TIR) domain protein *tir-1(RNAi)* animals in response to *P. aeruginosa* infection. L3 larvae were exposed to PA14 for 24 hours. Microscopic images are representatives from 3 independent experiments. (**B**) Quantification of reporter expression from data shown on panel (A) completed with data of control animals fed by OP50 for 24 h. (**C**) Quantification of SKN-1 nuclear translocation in *tir-1(RNAi)* L3 larvae upon 5 h PA14 exposure. Representative epifluorescence images of *tir(RNAi)* L3 larvae are shown in Figure S3. Please note that data in Figure 2B and 3C were derived from the same set of experiments. (**D**) Suggested model of SKN-1 activation during *P. aeruginosa* infection. Upon exposure to PA14, the TIR-1/PMK-1 pathway is indispensable but insufficient to elicit SKN-1 transactivation. We propose a second, unknown factor/pathway that is required to activate SKN-1. Whether the two pathways act in parallel or consecutively is unclear. Solid arrows indicate a direct, while dashed arrows indicate an indirect/unknown connection. EV: empty vector RNAi.

The p38 MAPK ortholog PMK-1 has a fundamental role in *C. elegans* innate immunity [11]. To investigate whether PMK-1 regulates SKN-1 in response to bacterial exposure, we monitored the activity of a *Pgcs-1*::GFP reporter in a wild-type and a *pmk-1(km25)* mutant genetic background (Figures 3A and 3B). We found that silencing *pmk-1* entirely prevented the SKN-1-dependent activation of *gcs-1* in response to PA14 infection. In physiological settings, PMK-1 is inactivated by the dual specificity MAPK phosphatase VHP-1 [23]. Suppression of VHP-1 resulted in increased PMK-1 phosphorylation and resistance to PA14 [23]. However, *vhp-1(RNAi)* significantly increased *Pgcs-1*::GFP activation upon PA14, but not upon OP50 exposure, suggesting that PMK-1 is an indispensable permissive factor for SKN-1 activation by infection.

TIR-1 is a conserved Toll/IL-1 resistance (TIR) domain protein known to activate p38 MAPK signaling independently of the Toll-like receptor ortholog *tol-1* during PA14 infection [24], [25]. Depletion of TIR-1 by RNAi prevented *Pgcs-1*::GFP fluorescence upon PA14 infection (Figures 3A and 3B). A similar inhibition in *Pgcs-1*::GFP expression was also observed in *tir-1(qd4)* mutant animals (data not shown). Moreover, silencing *tir-1* prevented the nuclear translocation of SKN-1 induced by PA14 infection, but did not affect its baseline expression levels (Figures 3C and S3.). Altogether, these results suggest that the TIR-1/PMK-1 pathway is necessary to attain activation of SKN-1 by PA14 exposure (Figure 3D).

### Involvement of SKN-1 in immunosenescence

**Figure 4:**
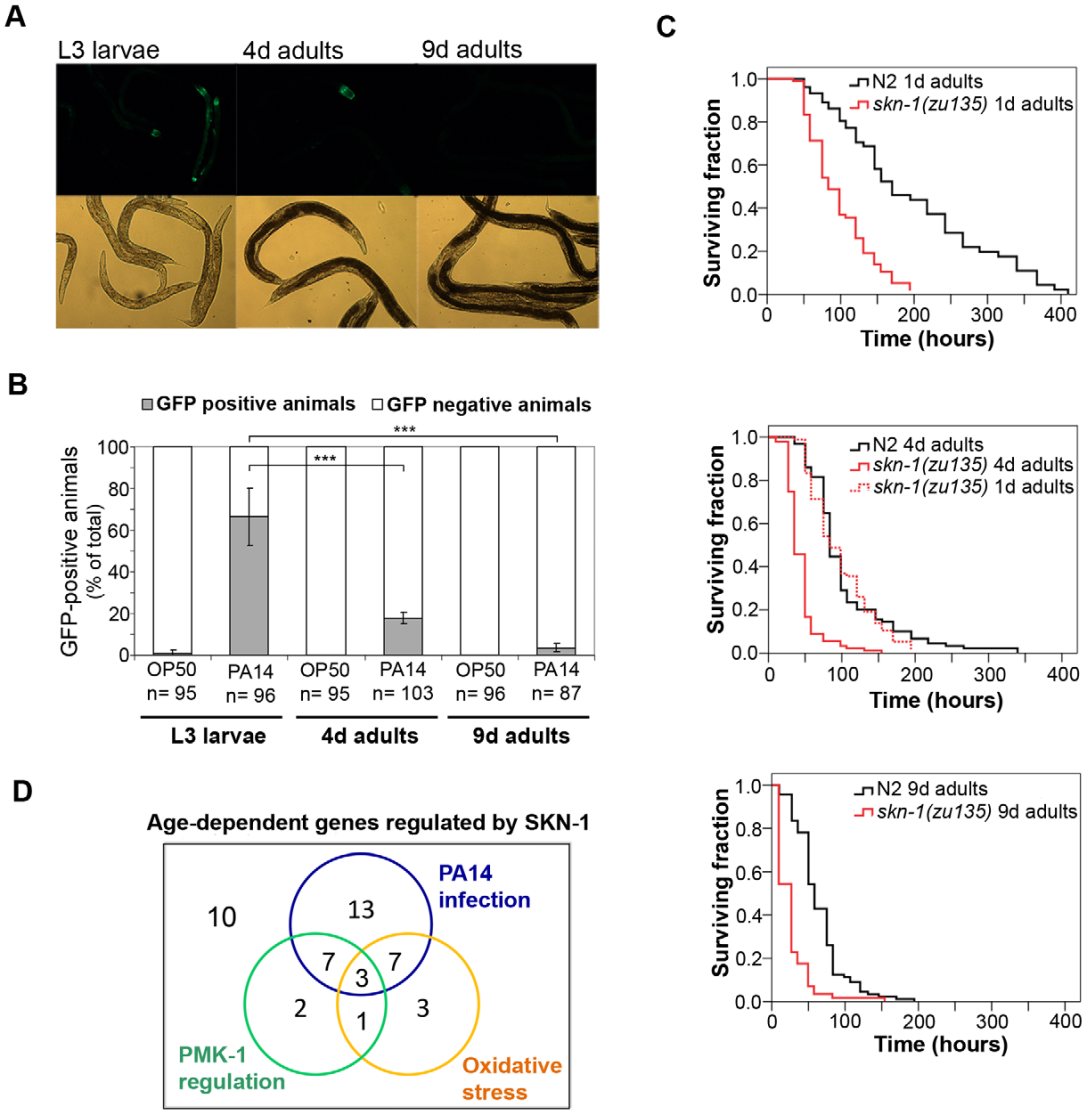
Involvement of SKN-1 in immunosenescence. (**A**) Representative epifluorescence images showing the decreased induction of the *gcs-1* promoter. L3 larvae, 4 d/9 d adult *Pgcs-1::gfp* worms were exposed to PA14 for 24 h. (**B**) Quantification of the epifluorescence images of panel (A). Epifluorescence images are representatives of two independent experiments. EV: empty vector RNAi. (**C**) Pathogen resistance of young adult (1 day-old), 4 day-old and 9 day-old adult N2 and *skn-1(zu135)* mutant animals. *skn-1(zu135)* mutant worms exhibited significantly increased susceptibility to PA14 compared to N2 wild-type animals at all ages (p<0.0001). 1 day-old adult *skn-1(zu135)* worms show similar pathogen resistance to 4 day-old N2 worms (p = 0.1429) (middle graph). Killing assays were performed with 3 parallel plates in each condition in 2 independent trials. (**D**) Venn diagram showing the distribution of age-regulated SKN-1 target genes. Data were analyzed by finding the overlaps between micro-array databases containing the genes down-regulated at least 10-fold in 15 d adult compared to 6 d adult wild-type animals [27] and SKN-1 dependent genes under non-stress [29] or oxidative stress conditions [30] using expression data from Wormbase [31]. Please note that the majority of genes belong to those regulated by PA14 infection. 10 of 46 genes could be assigned to none of the groups. For the detailed gene list please refer to Table S2.

Immune function declines with age, leading to compromised immune responses to infections in the elderly. Accordingly, aged nematodes exhibit increased susceptibility to infection by various pathogens, including *P. aeruginosa* [26]–[28]. As SKN-1 is required for both longevity and for pathogen resistance, we asked if chronological aging affected SKN-1-dependent target gene expression in nematodes exposed to pathogenic stress. To this end we examined the promoter induction of *gcs-1* by PA14 in L3 stage larvae, 4-day and in 9-day old adult worms, respectively (Figures 4A and 4B). We observed a massive age-dependent decrease in the expression of *Pgcs-1*::GFP reporter after 24 h of PA14 infection. To investigate, how SKN-1 activity is involved in immunosenescence, we examined the survival of 1, 4 and 9 day-old adult N2 and *skn-1(zu135)* mutant nematodes exposed to PA14. We observed that pathogen resistance in wild-type animals already declined at day 4 as previously described by Laws *et al.* [26]. Consistent with a premature decline of self defense in the absence of SKN-1 activity, 4 d adult N2 worms showed similar survival on PA14 to 1 d adult *skn-1(zu135)* animals (p = 0.1429). Furthermore, we found that *skn-1(zu135)* mutant animals exhibited increased susceptibility to PA14, compared to N2 at all ages (p>0.0001) (Figure 4C and Table S1B), indicating that SKN-1 function is also required to survive infection beyond day 9.

To address the potential involvement of SKN-1-dependent gene expression in immunosenescence, we performed a bioinformatics analysis using the microarray data of Youngman *et al.* [27]. From the 379 genes exhibiting the most significant down-regulation during aging (>10 fold down-regulation at d15 *vs.* d6) we identified 46 SKN-1-regulated genes (based on *skn-1(RNAi)* screens [29], [30]) (Figure 4D, Table S2). Next, we examined the regulation of these genes with respect to oxidative stress, PA14 and PMK-1 dependent regulation using Wormbase expression data [31]. Strikingly, SKN-1-regulated genes subject to PA14-dependent regulation were over-represented compared to those regulated by either oxidative stress or PMK-1, respectively. These results confirm a progressive age-dependent compromise in pathogen resistance and imply that a decline in SKN-1 function contributes to immunosenescence.

### Reduced IIS and oxidative preconditioning require SKN-1 for enhanced pathogen resistance

**Figure 5:**
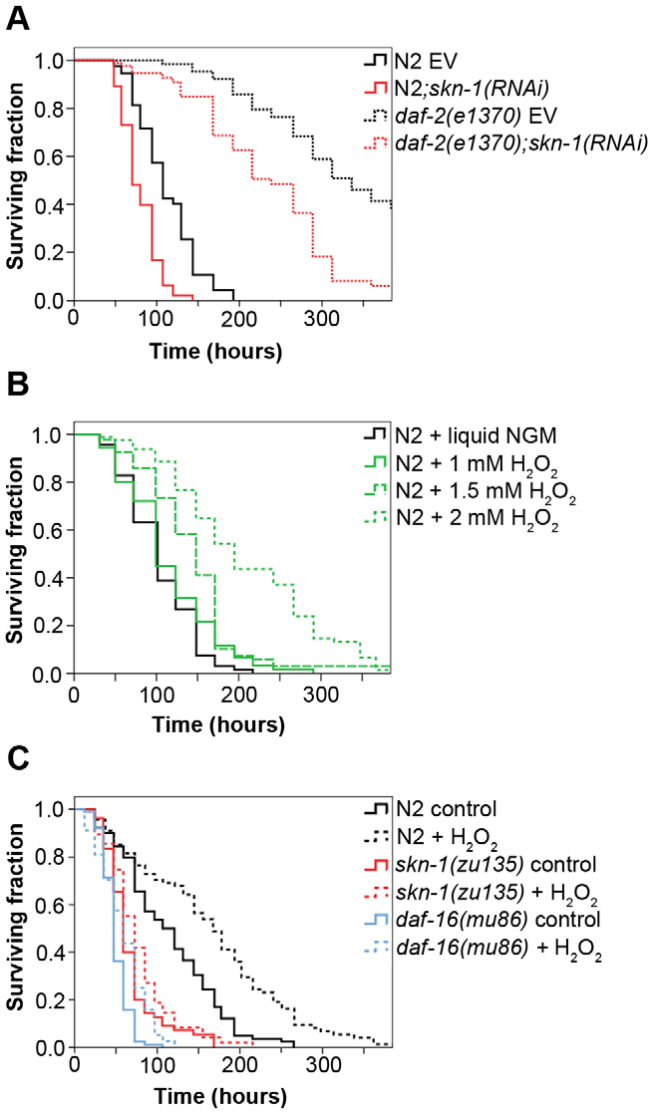
Reduced IIS and oxidative preconditioning require SKN-1 for enhanced pathogen resistance. (**A**) *daf-2(e1370)* mutant nematodes exhibited increased resistance to *P. aeruginosa*, compared to that of wild-type N2 worms (p<0.0001). *skn-1(RNAi)* treatment of *daf-2(e1370)* animals increased the susceptibility to *P. aeruginosa* infection (p<0.0001). Killing assays were performed with at least 90 young 1-day old adult animals in each condition. (**B**) H_2_O_2_ pretreatment increased survival on PA14 in a concentration-dependent manner. Survival curves of N2 wild-type worms treated with various concentrations of H_2_O_2_ in liquid NGM: 1 mM (p = 0.425), 1.5 mM (p<0.0001) and 2 mM (p<0.0001) 12 h prior to the killing assay are shown. Killing assay was performed with 90 3-day old adult animals in each condition. (**C**) Oxidative preconditioning-induced pathogen resistance was impaired in the absence of SKN-1 or DAF-16. Increase in survival was less pronounced in either *skn-1(zu135)* (p = 0.0156) or *daf-16(mu86)* mutant (p = 0.0304), than in wild-type animals (p<0.0001). Survival curves of the same genetic background were compared in the absence and presence of H_2_O_2_. Data were combined from at least two experiments with 89 animals in average for each group. EV: empty vector RNAi.

Loss-of-function mutations in the insulin/IGF-1 receptor gene, *daf-2* enhance stress resistance and extend lifespan, and both processes require DAF-16 and SKN-1 activity [15]. As reduced IIS increases pathogen resistance [6], we investigated the contribution of SKN-1 to pathogen resistance in *daf-2(e1370)* mutant animals. In accordance with previously published data [6], *daf-2(e1370)* mutants exhibited robustly increased pathogen resistance against PA14 (Figure 5A and Table S1C). However silencing *skn-1* by RNAi largely increased their susceptibility to PA14. These data suggest that SKN-1 is required for reduced IIS to bring about enhanced pathogen resistance against *P. aeruginosa*.

Exposure to mild oxidative stress induces tolerance to a lethal challenge, cross-tolerance to other stresses and extends lifespan [32]. To address the impact of oxidative preconditioning on pathogen resistance, nematodes were pretreated with various concentrations of H_2_O_2_, and then exposed to PA14 infection. H_2_O_2_ preconditioning induced resistance against PA14 in a concentration-dependent manner, reaching a 2-fold increase in survival by 2 mM H_2_O_2_, compared to untreated controls (Figure 5B and Table S1D). Intriguingly, the same treatment on *skn-1(zu135)* mutant nematodes not only exhibited a decreased pathogen resistance, but had a strongly suppressed reaction to H_2_O_2_ (Figure 5C and Table S1D). We also found that the mutation of another major oxidative stress response regulator, DAF-16 (*daf-16(mu86)*), shows an even shorter basal survival, compared to *skn-1(zu135)*, and poorly responded to H_2_O_2_ (Figure 5C and Table S1D). Thus, oxidative preconditioning requires both SKN-1 and DAF-16 for enhanced pathogen resistance against *P. aeruginosa*.

### Excessive activation of SKN-1 by *wdr-23(RNAi)* impairs pathogen resistance

**Figure 6:**
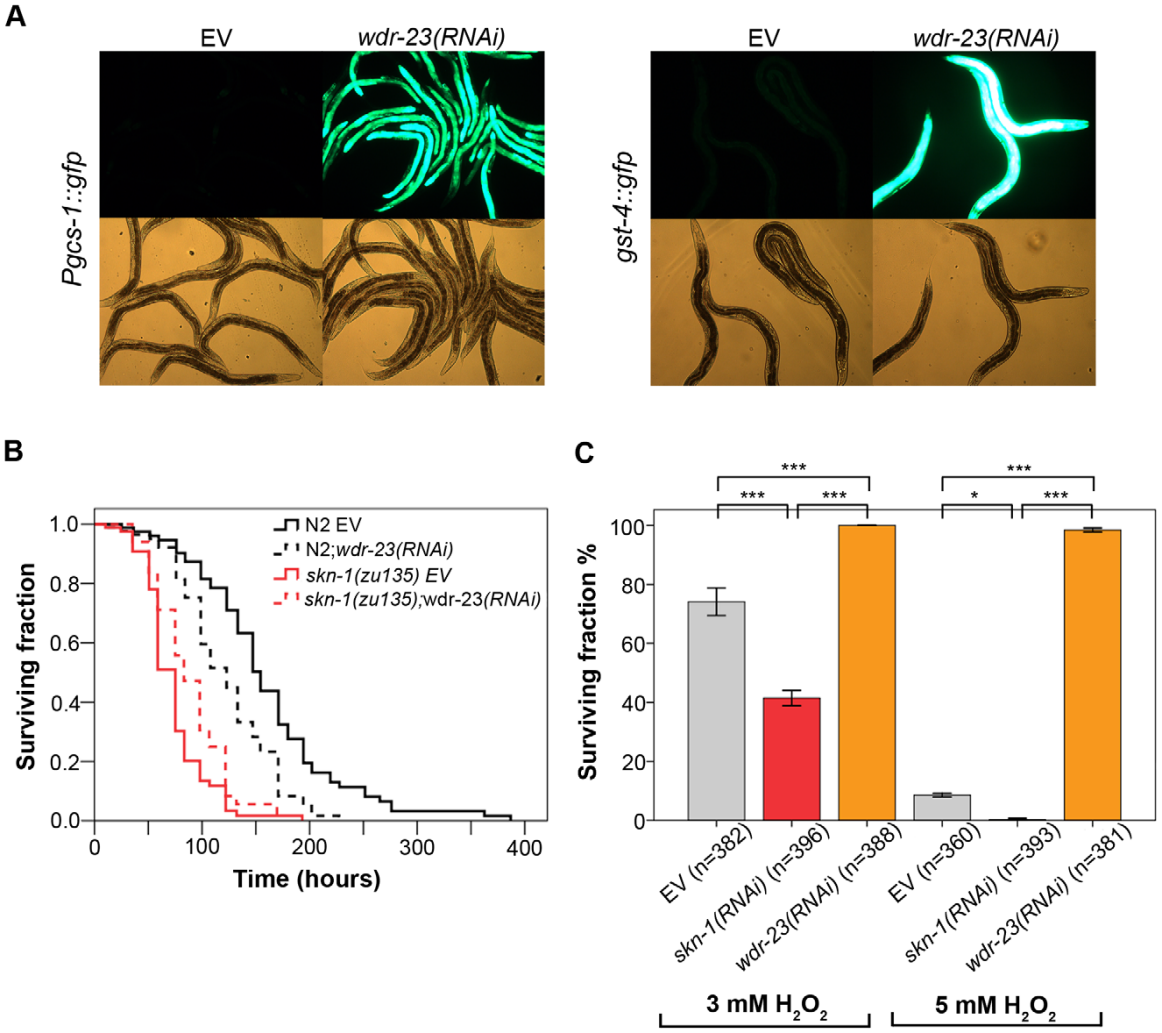
Excessive activation of SKN-1 by *wdr-23(RNAi)* impairs pathogen resistance. (**A**) Robust up-regulation of *Pgcs-1*::GFP and GST-4::GFP in 1 d adult *wdr-23(RNAi)* worms. (**B**) Pathogen resistance of *wdr-23(RNAi)*-fed N2 and *skn-1(zu135)* mutant worms. N2;*wdr-23(RNAi)* exhibited increased susceptibility to *P. aeruginosa* infection (p<0.0001). *skn-1(zu135)* mutant nematodes fed by *wdr-23(RNAi)* showed no significant difference in survival on PA14 (p = 0.1992). Killing assay was performed with at least 90 1-day old adult animals in each condition. Please note that data in Figures 1A and 6B were derived from the same set of experiments. (**C**) *wdr-23(RNAi)* treatment increased (p<0.0001, both at 3 mM and 5 mM H_2_O_2_, respectively), while *skn-1 RNAi* treatment decreased oxidative tolerance to H_2_O_2_ (p<0.0001 at 3 mM H_2_O, p<0.05 at 5 mM H_2_O_2_). Worms were treated with 3 mM or 5 mM H_2_O_2_ for 1 hour, and 24 h after challenge survival was scored. Data were combined from three experiments with 120 animals in average for each group. EV: empty vector RNAi.

Finally, we investigated whether increased activation of SKN-1 was able to promote pathogen resistance. Stabilization of SKN-1 by RNAi against *wdr-23* has been shown to induce constitutive SKN-1 activation, resistance to oxidative stress and longevity [19]. Feeding worms with *wdr-23(RNAi)* indeed resulted in an unexpectedly robust increase in the expression of *Pgcs-1*::GFP and GST-4::GFP (Figure 6A) compared to the PA14-induced expression (Figure 2C). To our surprise, *wdr-23(RNAi)*, compared to empty vector feeding greatly reduced pathogen resistance to PA14 (Figure 6B and Table S1A). *wdr-23(RNAi)* did not impair survival in a *skn-1(zu135)* mutant background excluding a SKN-1-independent impact of WDR-23 on pathogen resistance. Interestingly, the compromised reactivity of worms to *wdr-23(RNAi)* was confined to pathogenic stress. Determination of oxidative tolerance revealed that *wdr-23(RNAi)* animals exhibited increased survival, whereas *skn-1(RNAi)* nematodes displayed decreased survival, compared to control worms, when exposed to 3 mM or 5 mM H_2_O_2_, respectively (Figure 6C). Our results suggest that an excessive post-translational stabilization of SKN-1 induces oxidative stress resistance but impairs resistance to bacterial infection.

## Discussion

Our present study identified SKN-1 as a novel regulator of pathogen resistance against both Gram-negative *P. aeruginosa* and Gram-positive *E. faecalis* bacteria (Figures 1 and S1). We demonstrated a TIR-1/PMK-1-dependent SKN-1 activation upon *P. aeruginosa* infection (Figures 2 and 3). Moreover, we showed an early onset of immunosenescence with a parallel decline in SKN-1-dependent transcriptional activation (Figure 4) and a requirement of SKN-1 to efficient immunity in response to reduced IIS or preconditioning H_2_O_2_ treatment (Figure 5). Finally, we found that excessive activation of SKN-1 by blocking its turnover impaired pathogen resistance (Figure 6).

The xenobiotic stress response provides a conserved defense mechanism against oxidative and electrophilic stress via the induction of phase 2 detoxification enzymes [33]. Nrf2 and its nematode ortholog, SKN-1, are transcription factors important in oxidative and xenobiotic stress response [34], [35]. Previously, several studies demonstrated the importance of Nrf2 in innate immunity in mammals [36]–[38]. For example, Nrf2^−/−^ mice exhibit increased susceptibility to bacterial infection and bacterial lipopolysaccharide (LPS)-induced inflammation [39]. Similarly, our present study demonstrates that SKN-1 deficiency in *C. elegans* impairs resistance to infection (Figures 1 and S1). During the revision of our manuscript an independent paper from the Garsin lab appeared, which obtained similar results [21]. A previous study found no significant impairment of pathogen resistance by *skn-1(zu135)* and *skn-1(zu67)* mutations in the wildtype background [40]. A possible reason of this discrepancy might be the use of *cdc-25.1(RNAi)* by the Garsin lab and our study, suggesting that selective bagging in wild-type *vs.* sterile *skn-1* mutants might have masked the pathogen resistance decrease induced by loss of *skn-1* in the previous investigation. Consistently with this note, the use of *skn-1(RNAi)* from the L1 stage, which did not induce sterility [21], confirmed the decrease in pathogen resistance. A similar finding was also reported as an earlier unpublished result of Evans *et al.* [41].

We observed a nuclear translocation and transcriptional activation of SKN-1 in the intestine, consistent with the primary site of infection (Figures 2 and 3). Furthermore, we could not detect any apparent change in SKN-1 intensity or nuclear localization in ASI neurons upon PA14 exposure (Figure 2). This finding is in agreement with previous reports on constitutive SKN-1B activity and a lack of interaction between SKN-1B and WDR-23 in ASI neurons, respectively [19], [34]. Together, our data imply an active role of the intestinal SKN-1C isoform and does not allow a conclusion regarding the involvement of the ASI neuronal SKN-1B in the inducible antibacterial response. However, a continuous transcriptional output of SKN-1B and/or a different mode of regulation of SKN-1B in response to infection cannot be excluded. Hence, a tissue-specific analysis of SKN-1 function may give a clue whether SKN-1 isoforms co-operate in immunity.

Our findings confirm those of van der Hoeven *et al.* [21] on the critical role of the p38 MAPK pathway in SKN-1 activation (Figure 3). However, the inability of *vhp-1(RNAi)* to activate SKN-1 on OP50 suggests that there should be additional, unidentified signals that govern SKN-1 activation in response to PA14 infection, which will certainly prompt additional studies. We showed an absolute requirement of TIR-1 for SKN-1 nuclear translocation and for *gcs-1* promoter induction upon PA14 exposure, while the Garsin lab reported no to minimal involvement of TIR-1 in *gst-4* and *gcs-1* induction in response to *E. faecalis* [21]. Whether the difference between our observations beyond differences in assays and dosage/treatment by *tir-1(RNAi)* may be due to a differential pathogen sensing of *P. aeruginosa* and *E. faecalis* is an exciting possibility to explore. TIR-1 and PMK-1 are related to the mammalian SARM and p38 MAPK proteins, respectively [42], [43]. Although the existence of an orthologous pathway in mammals remains elusive, these findings indicate that the SKN-1-mediated response is an ancient component of innate immunity.

Immunosenescence, the age-dependent decline of immune response, is a critical problem impeding healthy ageing [44]. *C. elegans* provides a useful tool to investigate elements of innate immunity contributing to immunosenescence [45]. A recent systematic study reported an age-dependent progressive increase in susceptibility to PA14, detectable at day 6 of adulthood [27]. Our data on a similar age-related decline in survival, with a 45% decrease in pathogen resistance at day 4 (Figure 4C) establishes an earlier, dramatic onset of immunosenescence. Moreover, the loss of SKN-1 function phenocopies the decreased resistance of d4 worms already at day 1, and continues to negatively affect survival at day 9 (Figure 4C). A parallel strong decline in *gcs-1* transactivation on day 4 and the widespread down-regulation of SKN-1 targets, including PA14-regulated genes, between day 6 and 15 of adulthood (Figure 4D, Table S2) are consistent with this observation and indicate SKN-1 as a key player in immunosenescence.

Youngman and colleagues found an involvement of PMK-1 in a decline of the innate immune response, and hypothesized intestinal deterioration as a primary event in immunosenescence [27]. Of note, the dependence of SKN-1 activation on PMK-1 ([21] and our study), the high number of age-dependent SKN-1 targets among PMK-1 targets (13 of 26; Figure 4D, Table S2 and [27]) and the impact of SKN-1 on intestinal homeostasis [30] suggest a dynamic, probably mutual interaction between SKN-1 and PMK-1 in immunosenescence. We propose that SKN-1-dependent stress responses collapse early in adulthood, which manifest in a vicious circle of decreasing intestinal homeostasis, progressive immunosenescence and increasing pathogenic load in *C. elegans*. This hypothesis is consistent both with the short lifespan of worms in natural conditions and with the allocation of resources to maintain the soma until the production of fit progeny (the “disposable soma theory” [46]).

Genetic or environmental interventions that operate via stress-responsive mechanisms extend both lifespan and pathogen resistance [6], [15], [32], [47], [48]. Our results on H_2_O_2_-induced pathogen resistance (Figure 5B), together with previous analogous heat-shock experiments [7] suggest that mild stresses acting early in adulthood confer resistance against pathogenic stress. Furthermore, the demonstration of the requirement of SKN-1 and DAF-16 in the enhanced pathogen resistance of both H_2_O_2_-preconditioned and *daf-2(e1370)* mutant nematodes suggests a dynamic cross-talk of these stress-responsive transcription networks tipping the balance between responses to nutrient availability, oxidative and pathogen stress. Though our data do not allow a clear conclusion, the recent prediction of a DAF-16-dependent regulation of SKN-1 [49] is in line with the proposed functional interaction between SKN-1 and DAF-16 and is a subject of future interesting studies.

Evidence on mammals indicates a defensive role of Nrf2 against inflammation-induced tissue damage [36]–[39]. An analogous nematode model raises the question, whether SKN-1 affects immunity independently of its impact on aging. Indeed, longevity, stress resistance and pathogen resistance are intimately linked in short-lived *C. elegans*. However, a greater reduction of survival in *skn-1(zu135)* mutants on PA14 than on non-pathogenic OP50 (51% *vs.* 19% compared to N2, Figures 1 and S4, Tables S1 and S3) suggests a stronger impact of SKN-1 on pathogen resistance than on longevity. The pathogen-induced activation of SKN-1 and the large number of PA14-regulated SKN-1-targets including immune-related CUB-like domain proteins (Figure 4D, Table S2) [29] support SKN-1's active involvement in the pathogen response. Finally, it has previously been shown that knock-down of *skn-1* in the *daf-2(e1370)* mutant selectively suppresses stress resistance but not lifespan [15]. Thus, our findings demonstrating a SKN-1-dependent increase of pathogen resistance by this allele (Figure 5A), suggest an immune-specific effect of SKN-1. Studies investigating SKN-1-dependent responses on OP50 *vs.* pathogens would help reveal the downstream mediators of SKN-1 and to determine the immune-specific and other branches of SKN-1 action.

SKN-1 activation by *wdr-23(RNAi)* impairs pathogen resistance, a result in contrast with those of the Garsin lab [21]. The reason may lie in the use of *cdc-25.1(RNAi)* by us, or in the different dosage/duration of RNAi treatment in the two experimental protocols. Nevertheless, our findings on the adverse effects of excessive SKN-1 activity are consistent with those reporting that loss of WDR-23 activity slows growth via SKN-1 [19], expression of SKN-1 from high-copy arrays is toxic [15], and that SKN-1 mediates increased susceptibility to PA14 in the absence of BLI-3 [21]. Combining the two *wdr-23(RNAi)* results ([21] and our study) clearly shows that this type of activation can dissociate immunity from oxidative stress resistance. As a potential mechanism, excessive SKN-1 activation may remodel the transcriptional response in favor of anti-oxidative defense and/or may repress pathogen-specific defenses. Indeed, differential SKN-1 transcriptional outputs were demonstrated [29]. It is logical to assume that negative and positive inputs regulating SKN-1 allow fine-tuning of stress resistance, growth and immunity. An analogous deterioration of pathogen resistance by the excessive activation of DAF-16 [10] underscores the necessity of tight control of stress responses to avoid deleterious consequences during infection.

Taken together, we propose that an optimal enhancement of SKN-1 activity in proper time-frame may enhance immune responses and delay immunosenescence without compromising longevity. In recent years, *C. elegans* has become a versatile model not only for studying innate immunity, host-pathogen interactions, but for testing pharmacological interventions in drug discovery [50]. The results presented herein may prompt studies on drugs targeting SKN-1/Nrf2 to modulate the innate immune response. In conclusion, our findings indicate an intricate regulation of innate immunity by SKN-1, and link pathogenic stress signaling to xenobiotic and oxidative stress responses.

## Materials and Methods

### *C. elegans* strains and maintenance

Nematodes were maintained and propagated on *E. coli* OP50 as described by Brenner [51] at 20°C. The following *C. elegans* strains were obtained from the Caenorhabditis Genetics Center and were used in this study: N2, EU31 *skn-1(zu67)IV/nT1[unc-?(n754) let-?](IV;V)*, KU25 *pmk-1(km25)IV.*, ZD101 *tir-1(qd4)III*. Further strains were used: LD001 *Is007 [skn-1::gfp]*, CF1038 *daf-16(mu86)I.*, CB1370 *daf-2(e1370)III*. (Tibor Vellai, Eötvös Loránd University, Budapest, Hungary), LD1171 *Is003 [Pgcs-1::gfp]* (T. Keith Blackwell, Harvard Medical School, Boston MA, USA) and MJCU017 *kIs17[gst-4::gfp, pDP#MM016B]X*. (Johji Miwa, Chubu University, Kasugai, Japan). Nematodes were treated with *cdc-25.1(RNAi)* to avoid the bacterial infection induced ‘bag of worms’ phenotype in all experiment.

### Crossing and genotyping by PCR

*Pgcs-1::gfp;pmk-1(km25)* and *Pgcs-1::gfp;tir-1(qd4)* strains were created by mating male *pmk-1(km25)* or *tir-1(qd4)*, respectively, with LD1171 *Is003 [Pgcs-1::gfp]* hermaphrodites. Transgenic *rol* progeny was isolated with the correct genotype as scored by PCR. PCR primers were obtained from Sigma. The primers *pmk-1*-OF (5′-GGATACGGAAGAAGAGCCAATG-3′) and *pmk-1*-OR (5′-CAACAGTCTGCGTGTAATGC-3′) were used to detect the *pmk-1(km25)* deletion allele. The wild-type *pmk-1* allele amplified a 1195-bp fragment compared to a 882-bp fragment from *pmk-1(km25)* allele. Homozygous *pmk-1(km25)* mutants were identified by PCR using primers *pmk-1*-IF (5′-TCCTATAAGTTGCCATGACCTCAG-3′) and *pmk-1*-IR (5′-CCCGAGCGAGTACATTCAGC-3′) from inside the deletion region. Wild-type animals generated a 469-bp fragment, while the homozygous *pmk-1(km25)* allele did not produce any fragment. The primers *tir-1*-OF (5′-TGGGTAAATGAGGAAGAGAGAGAG-3′) and *tir-1*-OR (5′-TCGGTTGACGAGTCGAATTTGG-3′) were used to detect the *tir-1(qd4)* deletion allele. The wild-type *tir-1* allele amplified a 1368-bp fragment compared to a 228-bp fragment from *tir-1(qd4)* allele. Homozygous *tir-1(qd4)* mutants were identified by PCR using primers *tir-1*-OF and *tir-1*-IR (5′-CACAAGAACGTGCAACATCG-3′) from inside the deletion region. Wild-type animals generated a 327-bp fragment, while the homozygous *tir-1(qd4)* allele did not produce any fragment.

### RNA interference (RNAi)

The HT115(DE3) *E. coli* bacteria producing dsRNA against *cdc-25.1* (Andy Golden NIDDK/NIH, Bethesda MD, USA), *skn-1* (T. Keith Blackwell, Harvard Medical School, Boston MA, USA), *wdr-23* (Keith P. Choe, University of Florida, Gainesville FL, USA), *vhp-1* and *tir-1* (Source BioScience Geneservice, Cambridge, United Kingdom) were used in our study. RNAi feeding *E. coli* clones were grown overnight in LB medium containing 100 µg/ml ampicillin. RNAi treatment was performed as described by Shapira *et al.* [22]. Worms were grown on RNAi bacteria from hatching till young adult stage. If several RNAi constructs were used in one condition, ON cultures of the feeding bacteria strains were mixed equally. Empty vector containing HT115(DE3) bacteria (EV) was used as a control in all cases.

### Preparation of pathogenic bacteria

Different human opportunistic bacteria, such as *Pseudomonas aeruginosa* and *Enterococcus faecalis* [28], [52] are ubiquitously used as pathogen models. Gram-negative *Pseudomonas aeruginosa* PA14 (David W. Wareham, Queen Mary University of London, London, UK) and Gram-positive *Enterococcus faecalis* SdB262 (Jonathan J. Ewbank, Centre d'Immunologie de Marseille-Luminy, Marseille, France) bacteria were maintained and prepared for experiments as described by Powell and Ausubel [53]. BHI agar was supplemented with 100 µg/ml rifampicin for *E. faecalis* killing assay.

### Killing assay

Killing assays were performed with young adult animals at 25°C on slow killing plates (*P. aeruginosa*) or rifampicin BHI plates (*E. faecalis*), or otherwise as it was noted in the figure legend. Dead worms were scored every 12 hours till complete extinction of the population. Viability was determined by assaying for movement in response to gentle prodding. Worms died on the wall of the Petri dish or crawled into the gel were censored. 30 animals per condition were tested with 3 parallel plates in at least two independent trials, except that an H_2_O_2_-concentration dependence of preconditioned pathogen resistance was established in one trial. For studying pathogen resistance of *daf-2* mutants, nematodes were grown at 15°C. For oxidative preconditioning 2-day old adult animals were treated with 0 (control), 1 mM, 1.5 mM and 2 mM H_2_O_2_ (Sigma) in liquid NGM for 2 hours at 20°C. Before the killing assay worms were transferred to OP50 seeded NGM plates for a 12-hour recovery period. To test the effect of aging on pathogen resistance, worms were maintained on OP50-seeded NGM plates before the challenge. To avoid ‘bag of worms’ phenotype, animals were fed by *cdc-25.1(RNAi)*.

### Fluorescence microscopy

Nematodes were treated as indicated in the figure legends (Figures 2, 3, 4 and 6). After treatments at least 40 worms per condition were placed on a 2% agarose pad, and immobilized by adding 40 mM levamisole in M9 buffer. Images were taken by a Leica DMI6000B epifluorescence microscope with a DFC480 camera. Epifluorescent microscopic images are representatives of at least 3 experiments. To analyze *Pgcs-1*::GFP and GST-4::GFP expression upon PA14 infection, two groups of animals were determined depending on the detected GFP level in the intestine: GFP positive and GFP negative animals. To study the nuclear localization of SKN-1 in response to PA14 infection minimum 15 *skn-1::gfp* worms per condition were analyzed in at least 3 independent trials. Images were captured by a Zeiss LSM510 confocal laser scanning microscope equipped with a 40×/1.3 oil immersion objective (Plan-Neofluar, Zeiss).

### Analysis of SKN-1 dependent targets amongst genes down-regulated by aging

A list of 379 genes exhibiting the most significant age-dependent decline in their expression (>10-fold at d6 *vs.* d15) was acquired from [27]. Data were analyzed by finding the overlaps between genes subject to SKN-1 dependent genes under non-stress [29] or oxidative stress conditions [30]. Then the expression of the identified genes was analyzed based on Wormbase data, focusing on PA14-, oxidative stress- or PMK-1-dependent regulation [31].

### Oxidative stress tolerance

Young, 1-day old adult worms were incubated in liquid NGM for 1 hour at 20°C with 3 mM and 5 mM H_2_O_2_ (Sigma). After oxidative challenge animals were transferred to OP50 seeded NGM plates and viability was tested 24 hours later. 35 animals per plate were examined in each condition with 3 parallel plates in 3 independent trials.

### Statistical analysis

Data were analyzed by using the SPSS software 15.0 (SPSS Inc., Chicago, IL, USA). Survival curves were compared by Kaplan-Meyer log-rank test. To compare the means of survival (oxidative tolerance assay) or the GFP expression of the *Pgcs-1::gfp, gst-4::gfp, skn-1::gfp* strains variables were analyzed by one-way ANOVA test. Results are expressed as mean ± standard deviation (SD). Statistical significance was indicated as follows: * p<0.05, ** p<0.001, *** p<0.0001.

### Accession numbers

*C. elegans* proteins/genes: Q17941, Q9XTG7, Q2MGF0, Q17450, O61213, P54145, Q9U3Q6, O02215, G5EC10, Q18198, Q9XUH3, Q9XUF9, O44552, Q27487, Q18938, O17725, O16849, Q968Y9, Q9XVB4, Q9XVA9, Q19223, O62146, G5EGH6, Q8MNR8, Q19774, O02357, Q09321, Q9UAQ9, P91316, Q20770, Q20840, G5EC22, P90893, Q20968, Q21009, Q20117, Q9U2Q9, Q21355, Q9XW45, Q21381, Q09991, Q94269, Q94271, P34528, Q17446, Q2PJ68, P34707, P41977, O02364, Q9XUC0, Q86DA5, Q10038, P90794, Q8WRF1, Q9U309, G5EFR9, Q9GR66, G5ECJ8, Q86S61, O76725, Q9N4X8, Q9NAB1, Q23498, Q23564

Human proteins/genes: Q12778, Q16656, Q16236, Q9Y4A8

## Supporting Information

Figure S1 **SKN-1 is required for pathogen resistance against both** ***P. aeruginosa*** **and** ***E. faecalis*** **.**
(DOC)

Figure S2 **PA14-induced activation of** ***Pgcs-1*** **::GFP and GST-4::GFP expression.**
(DOC)

Figure S3 **Suppression of PA14-induced SKN-1 nuclear localization by** ***tir-1(RNAi)*** **.**
(DOC)

Figure S4 **Lifespan of N2 and** ***skn-1(zu135)*** **mutant worms.**
(DOC)

Table S1 **Statistical analysis of killing assays.**
(DOC)

Table S2 **List of SKN-1-dependent genes down-regulated by aging.**
(DOC)

Table S3 **Statistical analysis of lifespan assays.**
(DOC)
