# Supplementary material for: A Role for SKN-1/Nrf in Pathogen Resistance and Immunosenescence in Caenorhabditis elegans
Source: PLoS Pathog. 2012 Apr 26;8(4):e1002673. doi: 10.1371/journal.ppat.1002673 (PMC3343120; doi:10.1371/journal.ppat.1002673)
Supplement: Figure S1 — SKN-1 is required for pathogen resistance against both P. aeruginosa and E. faecalis . (DOC) [file ppat.1002673.s001.doc]

**Figure S1**


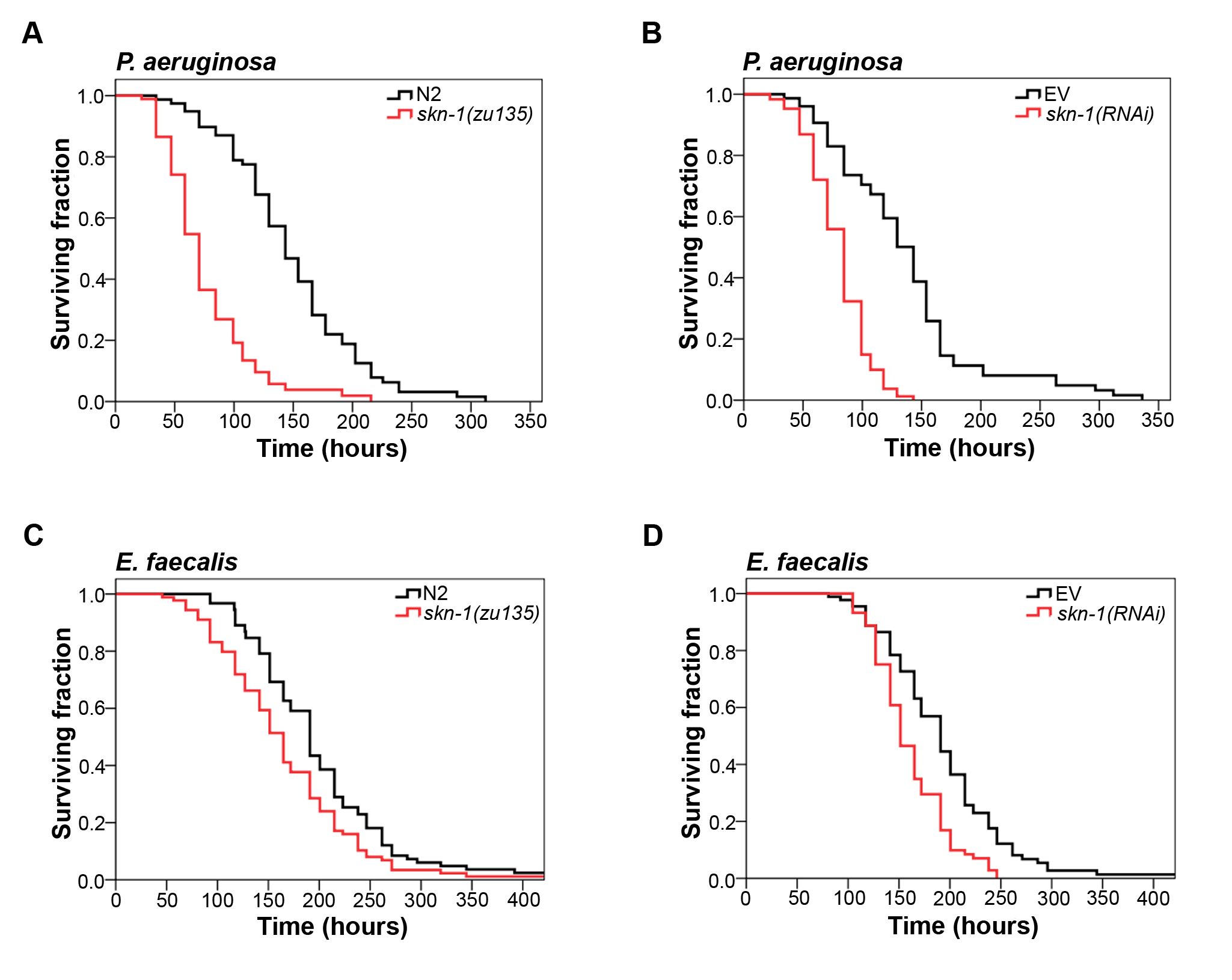


**Figure S1. SKN-1 is required for pathogen resistance against both *P. aeruginosa* and *E. faecalis* (A, B)** Increased susceptibility to Gram-negative *Pseudomonas aeruginosa* PA14 occurs in both *skn-1(zu135)* mutant (p<0.0001) and *skn-1(RNAi)* nematodes (p<0.0001). **(C, D)** Increased susceptibility to Gram-positive *Enterococcus faecalis* SdB262 occurs in both *skn-1(zu135)* (p<0.001) and *skn-1(RNAi)* (p<0.0001) animals. Killing assays were performed with at least 90 2-day old adult animals in each condition. EV: empty vector RNAi.
