# Supplementary material for: A Role for SKN-1/Nrf in Pathogen Resistance and Immunosenescence in Caenorhabditis elegans
Source: PLoS Pathog. 2012 Apr 26;8(4):e1002673. doi: 10.1371/journal.ppat.1002673 (PMC3343120; doi:10.1371/journal.ppat.1002673)
Supplement: Figure S2 — PA14-induced activation of Pgcs-1 ::GFP and GST-4::GFP expression. (DOC) [file ppat.1002673.s002.doc]

**Figure S2**


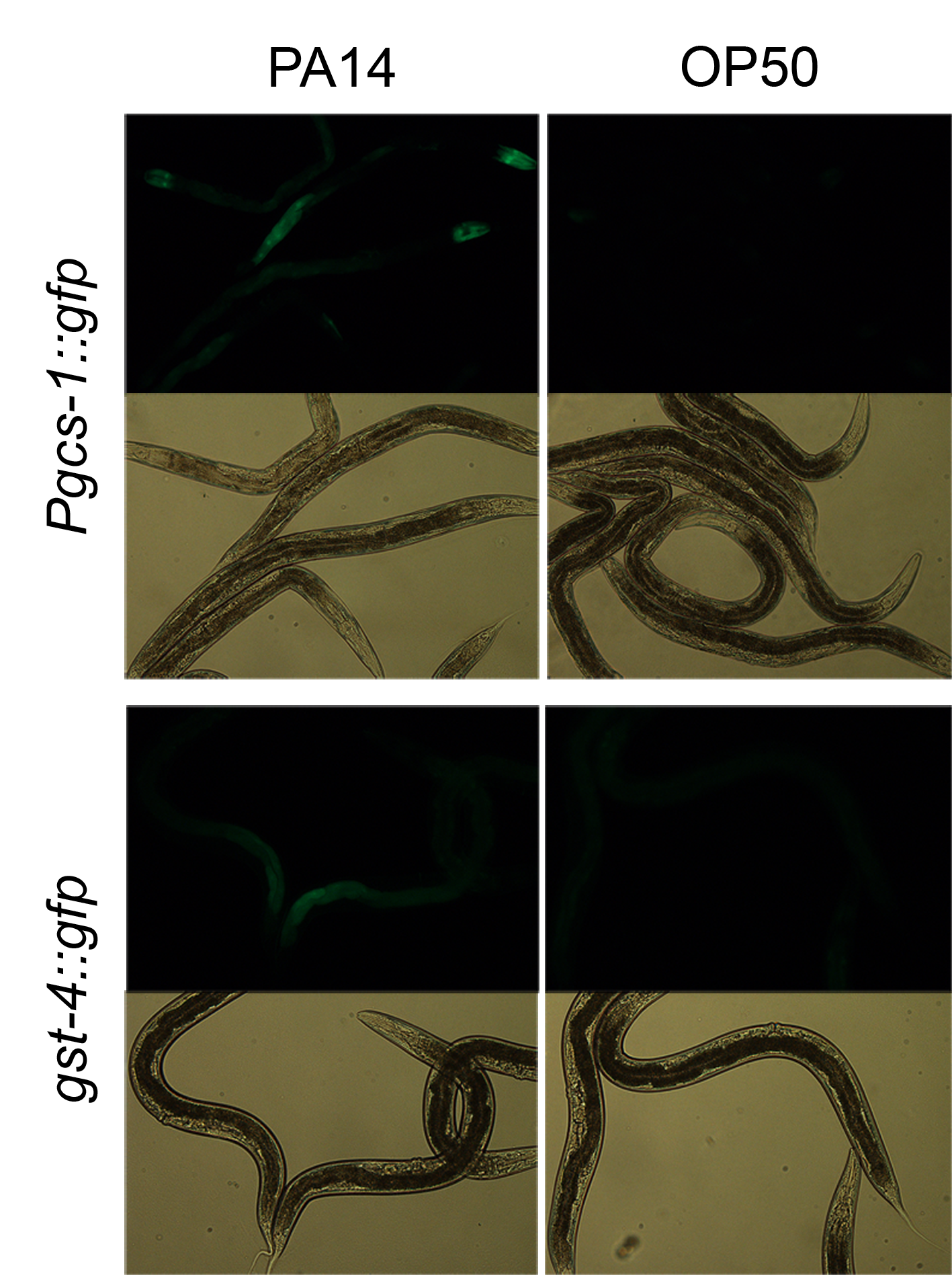


**Figure S2. PA14-induced activation of *Pgcs-1::*GFP and GST-4::GFP expression** Representative epifluorescence microscopic image showing intestinal expression of *Pgcs-1::*GFP and GST-4::GFP in L3 larvae upon a 24-hour PA14 exposure compared to the OP50 control. This figure supplements the images of animals exposed to PA14 on Fig. 2C.
