# Supplementary material for: A Role for SKN-1/Nrf in Pathogen Resistance and Immunosenescence in Caenorhabditis elegans
Source: PLoS Pathog. 2012 Apr 26;8(4):e1002673. doi: 10.1371/journal.ppat.1002673 (PMC3343120; doi:10.1371/journal.ppat.1002673)
Supplement: Figure S3 — Suppression of PA14-induced SKN-1 nuclear localization by tir-1(RNAi) . (DOC) [file ppat.1002673.s003.doc]

**Figure S3**


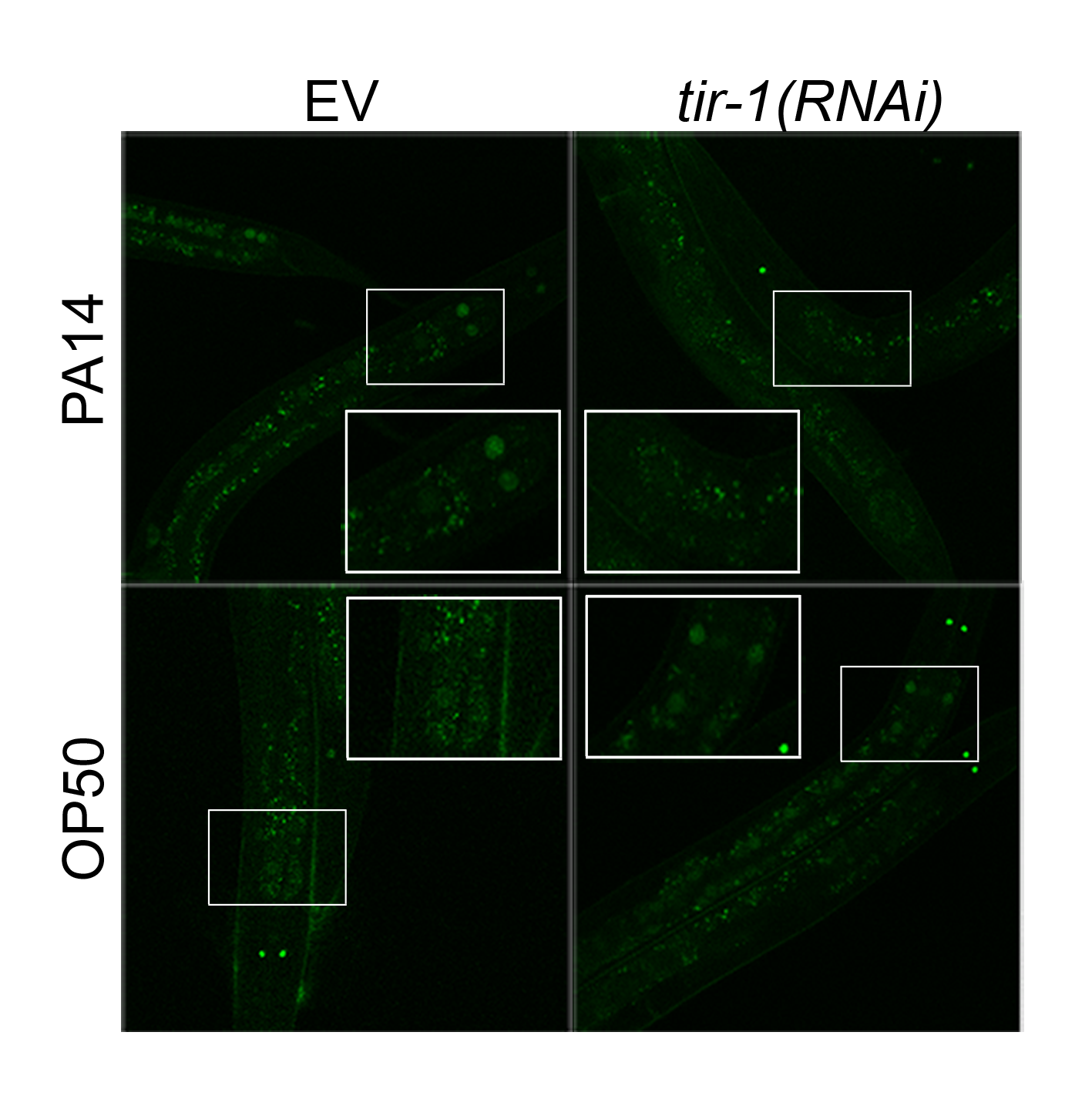


**Figure S3. Suppression of PA14-induced SKN-1 nuclear localization by *tir-1(RNAi)*** Representative epifluorescence images showing SKN-1::GFP nuclear translocation in the *Is007[*SKN-1::GFP*]* strain fed by empty vector or *tir-1(RNAi)*. L3 larvae were exposed to *P. aeruginosa* PA14 for 5 hours. Quantification of SKN-1 nuclear translocation data is shown on Figure 3C.
