# Supplementary material for: A Role for SKN-1/Nrf in Pathogen Resistance and Immunosenescence in Caenorhabditis elegans
Source: PLoS Pathog. 2012 Apr 26;8(4):e1002673. doi: 10.1371/journal.ppat.1002673 (PMC3343120; doi:10.1371/journal.ppat.1002673)
Supplement: Figure S4 — Lifespan of N2 and skn-1(zu135) mutant worms. (DOC) [file ppat.1002673.s004.doc]

**Figure S4**


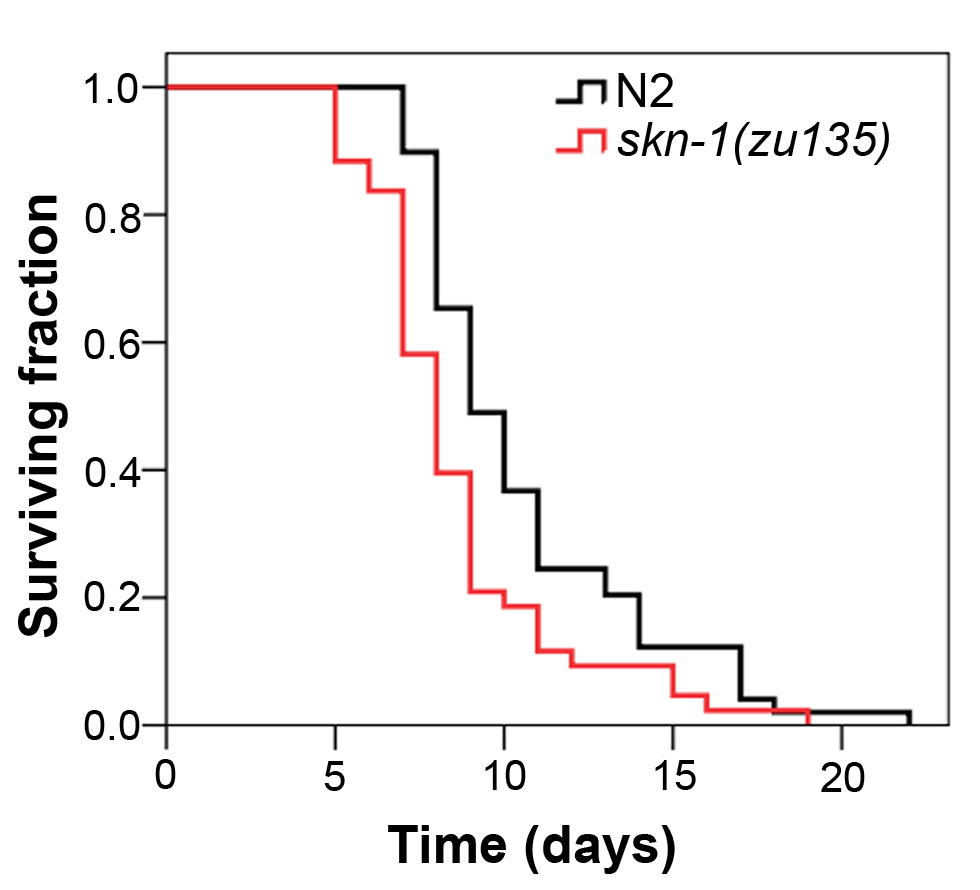


**Figure S4. Lifespan of N2 and *skn-1(zu135)* mutant worms** *skn-1(zu135)* mutant worms exhibit shortened lifespan compared to N2 worms (p=0.0048). Nematodes were fed by *cdc-25.1(RNAi)* to preserve the same experimental conditions used in killing assays. Lifespan experiments were performed on OP50 seeded NGM plates with at least 2 parallel plates with 25 animals in each condition in 3 independent trials. Animals were scored dead or censored with the same process as described in Materials and Methods at killing assays.
