# Supplementary material for: A Role for SKN-1/Nrf in Pathogen Resistance and Immunosenescence in Caenorhabditis elegans
Source: PLoS Pathog. 2012 Apr 26;8(4):e1002673. doi: 10.1371/journal.ppat.1002673 (PMC3343120; doi:10.1371/journal.ppat.1002673)
Supplement: Table S1 — Statistical analysis of killing assays. (DOC) [file ppat.1002673.s005.doc]

**Table S1: Statistical analysis of killing assays**

**A. Survival data of N2, *skn-1(zu135)* mutants and N2;*skn-1(RNAi)* worms with or without *wdr-23(RNAi)* treatment exposed to *P. aeruginosa***

| **Figure** | **Pathogen,**  **trial** | **Strains and conditions** | **Mean survival (hours)** | **N (total)** | **N (dead)** | **% change *vs.* N2; EV** | **p *vs.* N2;EV (log rank)** | **% change *vs. skn-1(zu135) or skn-1(RNAi)*** | **p *vs.* *skn-1(zu135)* or**  ***skn-1(RNAi)***  **(log rank)** |
| --- | --- | --- | --- | --- | --- | --- | --- | --- | --- |
| 1A and 6B | PA14, 1 | N2; EV | 160.7 | 92 | 64 |  |  |  |  |
|  |  | N2;*wdr-23(RNAi)* | 122.0 | 92 | 63 | -24.1 | <0.0001 | 66.4 | <0.0001 |
|  |  | *skn-1(zu135);* EV | 73.3 | 90 | 63 | -54.4 | <0.0001 |  |  |
|  |  | *skn-1(zu135);* *wdr-23(RNAi)* | 88.9 | 82 | 46 | -44.7 | <0.0001 | 21.2 | 0.1992 |
|  | PA14, 2 | N2; EV | 147.7 | 91 | 77 |  |  |  |  |
|  |  | N2;*wdr-23(RNAi)* | 113.4 | 93 | 77 | -23.2 | 0.0002 | 51.3 | <0.0001 |
|  |  | *skn-1(zu135);* EV | 75.0 | 98 | 76 | -49.3 | <0.0001 |  |  |
|  |  | *skn-1(zu135);* *wdr-23(RNAi)* | 86.3 | 95 | 67 | -41.6 | <0.0001 | 15.1 | 0.47 |
| 1B and 6B | PA14, 1 | N2; EV | 127.3 | 90 | 68 |  |  |  |  |
|  |  | N2 *skn-1(RNAi)* | 79.0 | 90 | 57 | -37.9 | <0.0001 |  |  |
|  |  | N2 *wdr-23(RNAi*) | 88.3 | 93 | 84 | -30.7 | <0.0001 | 11.7 | 0.0778 |
|  | PA14, 2 | N2; EV | 150.6 | 90 | 73 |  |  |  |  |
|  |  | N2 *skn-1(RNAi)* | 126.9 | 105 | 84 | -15.7 | 0.0013 |  |  |
|  |  | N2 *wdr-23(RNAi*) | 141.5 | 90 | 81 | -6.0 | 0.0439 | 11.5 | 0.5619 |

**B. Survival data of aged N2 and *skn-1(zu135)* animals upon exposure to *P. aeruginosa***

| **Figure** | **Pathogen,**  **trial** | **Strains and conditions** | **Mean survival (hours)** | **N (total)** | **N (dead)** | **% change *vs.* N2 young adults** | **p *vs.***  **N2 young adults**  **(log rank)** | **% change *vs.* s*kn-1(zu135)* young adults** | **p *vs.***  **s*kn-1(zu135) young adults***  **(log rank)** | **% change *vs.* N2 of identical age** | **p *vs.* N2 of identical age**  **(log rank)** |
| --- | --- | --- | --- | --- | --- | --- | --- | --- | --- | --- | --- |
| 4C | PA14, 1 | N2 young adults | 198.6 | 95 | 54 |  |  |  |  |  |  |
|  |  | N2 4 day-old adults | 104.5 | 102 | 90 | -47.4 | <0.0001 | 6.5 | 0.1429 |  |  |
|  |  | N2 9 day-old adults | 64.4 | 91 | 89 | -67.6 | <0.0001 | -34.3 | <0.0001 |  |  |
|  |  | *skn-1(zu135)* young adults | 98.1 | 106 | 75 | -50.6 | <0.0001 |  |  |  |  |
|  |  | *skn-1(zu135)* 4 day-old adults | 44.6 | 95 | 91 | -77.5 | <0.0001 | -54.5 | <0.0001 | -57.3 | <0.0001 |
|  |  | *skn-1(zu135)* 9 day-old adults | 26.0 | 57 | 57 | -86.9 | <0.0001 | -73.5 | <0.0001 | -59.6 | <0.0001 |
|  | PA14, 2 | N2 young adults | 186.5 | 90 | 57 |  |  |  |  |  |  |
|  |  | N2 4 day-old adults | 107.3 | 91 | 77 | -42.5 | <0.0001 | 10.6 | 0.3203 |  |  |
|  |  | N2 9 day-old adults | 58.1 | 95 | 92 | -68.9 | <0.0001 | -40.1 | <0.0001 |  |  |
|  |  | *skn-1(zu135)* young adults | 97.0 | 91 | 71 | -48.0 | <0.0001 |  |  |  |  |
|  |  | *skn-1(zu135)* 4 day-old adults | 48.4 | 92 | 83 | -74.1 | <0.0001 | -50.1 | <0.0001 | -54.9 | <0.0001 |
|  |  | *skn-1(zu135)* 9 day-old adults | 25.5 | 80 | 80 | -86.4 | <0.0001 | -73.7 | <0.0001 | -56.2 | <0.0001 |

**C. Survival data of *daf-2(e1370)* IIS mutants fed by *skn-1(RNAi)* upon exposure to *P. aeruginosa***

| **Figure** | **Pathogen.**  **trial** | **Strains and conditions** | **Mean survival (hours)** | **N (total)** | **N (dead)** | **% change *vs.* N2; EV** | **p *vs.* N2; EV (log rank)** | **% change *vs.* *daf-2(e1370)*;**  **EV** | **p *vs.* *daf-2(e1370)*;**  **EV**  **(log rank)** |
| --- | --- | --- | --- | --- | --- | --- | --- | --- | --- |
| 5A | PA14, 1 | N2; EV | 111.4 | 90 | 51 |  |  |  |  |
|  |  | N2 *skn-1(RNAi)* | 79.5 | 91 | 53 | -28.7 | <0.0001 |  |  |
|  |  | *daf-2(e1370);* EV | 310.0 | 100 | 39 | 178.2 | <0.0001 |  |  |
|  |  | daf-2(e1370);*skn-1(RNAi)* | 231.7 | 91 | 48 | 107.9 | <0.0001 | -25.3 | <0.0001 |
|  | PA14, 2 | N2; EV | 108.5 | 102 | 73 |  |  |  |  |
|  |  | N2 *skn-1(RNAi)* | 78.4 | 101 | 63 | -27.8 | <0.0001 |  |  |
|  |  | *daf-2(e1370);* EV | 288.7 | 114 | 35 | 166.0 | <0.0001 |  |  |
|  |  | daf-2(e1370);*skn-1(RNAi)* | 262.7 | 124 | 36 | 142.1 | <0.0001 | -9.0 | 0.0098 |
|  | PA14, 3 | N2; EV | 132.6 | 97 | 73 |  |  |  |  |
|  |  | N2 *skn-1(RNAi)* | 78.5 | 104 | 68 | -40.8 | <0.0001 |  |  |
|  |  | *daf-2(e1370);* EV | 277.4 | 131 | 46 | 109.3 | <0.0001 |  |  |
|  |  | daf-2(e1370);*skn-1(RNAi)* | 160.4 | 105 | 44 | 21.0 | 0.1194 | -42.2 | <0.0001 |

***D. Survival data of H2O2-preconditioned N2, skn-1(zu135) and daf-16(mu86) mutants exposed to*** P. aeruginosa

| **Figure** | **Pathogen,**  **Trial** | **Strains and conditions** | **Mean survival (hours)** | **N (total)** | **N (dead)** | **% change *vs.* N2 ctr** | **p *vs.* N2 ctr (log rank)** | **% change H_2_O_2_ *vs.* unconditioned controls** | **p H_2_O_2_ *vs.* unconditioned controls**  **(log rank)** |
| --- | --- | --- | --- | --- | --- | --- | --- | --- | --- |
| 5B | PA14, 1 | N2 ctr | 103.9 | 90 | 71 |  |  |  |  |
|  |  | N2 + 1 mM H2O2 | 115.6 | 90 | 63 | 11.2 | 0.4253 |  |  |
|  |  | N2 + 1.5 mM H2O2 | 145.7 | 90 | 71 | 40.2 | <0.0001 |  |  |
|  |  | N2 + 2 mM H2O2 | 209.7 | 90 | 76 | 101.8 | <0.0001 |  |  |
| 5C | PA14, 1 | N2 ctr | 115.1 | 90 | 84 |  |  |  |  |
|  |  | N2 + 2mM H2O2 | 165.6 | 90 | 79 | 43.9 | <0.0001 |  |  |
|  |  | *skn-1(zu135)* ctr | 67.4 | 90 | 58 | -41.4 | <0.0001 |  |  |
|  |  | skn-1(zu135) + 2mM H2O2 | 77.4 | 90 | 57 | -32.7 | <0.0001 | 14.9 | 0.0156 |
|  |  | *daf-16(mu86)* ctr | 49.7 | 90 | 85 | -56.8 | <0.0001 |  |  |
|  |  | daf-16(mu86) + 2mM H2O2 | 50.5 | 90 | 84 | -56.1 | <0.0001 | 1.6 | 0.0304 |
|  | PA14, 2 | N2 ctr | 130.7 | 90 | 58 |  |  |  |  |
|  |  | N2 + 2mM H2O2 | 181.7 | 90 | 76 | 39.1 | <0.0001 |  |  |
|  |  | *skn-1(zu135)* ctr | 87.6 | 90 | 72 | -33.0 | <0.0001 |  |  |
|  |  | skn-1(zu135) + 2mM H2O2 | 98.0 | 90 | 62 | -25.0 | 0.0079 | 12.0 | 0.1744 |
|  | PA14, 3 | N2 ctr | 91.6 | 90 | 60 |  |  |  |  |
|  |  | N2 + 2mM H2O2 | 155.3 | 90 | 74 | 69.5 | <0.0001 |  |  |
|  |  | *daf-16(mu86)* ctr | 53.9 | 90 | 77 | -41.2 | <0.0001 |  |  |
|  |  | daf-16(mu86) + 2mM H2O2 | 52.8 | 78 | 70 | -42.4 | 0.0012 | -2.0 | 0.0526 |

**E. Survival data of 2d adult *skn-1(zu135)* mutants and *skn-1(RNAi)* worms exposed to *P. aeruginosa* and *E. feacalis***

| **Figure** | **Pathogen, trial** | **Strains and conditions** | **Mean survival (hours)** | **N (total)** | **N (dead)** | **% change *vs.* N2 or N2; EV** | **p *vs.* N2 or N2; EV (log rank)** |
| --- | --- | --- | --- | --- | --- | --- | --- |
| S1A | PA14, 1 | N2 | 148.5 | 90 | 68 |  |  |
|  |  | *skn-1(zu135)* | 76.5 | 90 | 55 | -48.5 | <0.0001 |
|  | PA14, 2 | N2 | 126.4 | 90 | 86 |  |  |
|  |  | *skn-1(zu135)* | 88.4 | 90 | 85 | -30.1 | <0.0001 |
|  | PA14, 3 | N2 | 165.5 | 90 | 73 |  |  |
|  |  | *skn-1(zu135)* | 120.9 | 90 | 70 | -27.0 | 0.0002 |
| S1B | PA14, 1 | N2; EV | 136.9 | 90 | 64 |  |  |
|  |  | *N2 skn-1(RNAi)* | 80.3 | 120 | 85 | -41.4 | <0.0001 |
|  | PA14, 2 | N2; EV | 142.5 | 90 | 87 |  |  |
|  |  | *N2 skn-1(RNAi)* | 80.0 | 90 | 78 | -43.8 | <0.0001 |
|  | PA14, 3 | N2; EV | 145.6 | 90 | 80 |  |  |
|  |  | *N2 skn-1(RNAi)* | 97.3 | 90 | 71 | -33.2 | <0.0001 |
| S1C | *E. faecalis* SdB262, 1 | N2 | 180.6 | 91 | 87 |  |  |
|  |  | *skn-1(zu135)* | 131.3 | 90 | 81 | -27.3 | <0.0001 |
|  | *E. faecalis* SdB262, 2 | N2 | 200.7 | 94 | 86 |  |  |
|  |  | *skn-1(zu135)* | 166.1 | 91 | 88 | -17.2 | 0.0009 |
|  | *E. faecalis* SdB262, 3 | N2 | 179.3 | 90 | 78 |  |  |
|  |  | *skn-1(zu135)* | 133.4 | 90 | 80 | -25.6 | <0.0001 |
| S1D | *E. faecalis* SdB262, 1 | N2; EV | 173.8 | 91 | 87 |  |  |
|  |  | *N2 skn-1(RNAi)* | 133.8 | 90 | 87 | -23.0 | <0.0001 |
|  | *E. faecalis* SdB262, 2 | N2; EV | 193.7 | 90 | 80 |  |  |
|  |  | *N2 skn-1(RNAi)* | 161.3 | 91 | 80 | -16.7 | <0.0001 |
|  | *E. faecalis* SdB262, 3 | N2; EV | 198.6 | 90 | 82 |  |  |
|  |  | *N2 skn-1(RNAi)* | 160.8 | 90 | 80 | -19.0 | <0.0001 |

N (total) (total number of observations) = N (dead) + N (censored).
