# Supplementary material for: A Role for SKN-1/Nrf in Pathogen Resistance and Immunosenescence in Caenorhabditis elegans
Source: PLoS Pathog. 2012 Apr 26;8(4):e1002673. doi: 10.1371/journal.ppat.1002673 (PMC3343120; doi:10.1371/journal.ppat.1002673)
Supplement: Table S2 — List of SKN-1-dependent genes down-regulated by aging. (DOC) [file ppat.1002673.s006.doc]

**Table S2: List of SKN-1-dependent genes down-regulated by aging^#^**

| **Sequence name** | **Gene**  **name** | **PA14 infection^*^** | **Oxidative stress^*^** | **Regulation by PMK-1^*^** | **Reference^$^** |
| --- | --- | --- | --- | --- | --- |
| F01D5.5 |  | **+** | **+** | **+** | Oliveira *et al.* [3] |
| F54D5.4 |  | **+** | **+** | **+** | Oliveira *et al.* [3] |
| Y39G10AR.6 | *ugt-31* | **+** | **+** | **+** | Oliveira *et al.* [3] |
| B0213.15 | *cyp-34A9* | **+** | **+** |  | Park *et al.* [4] |
| F09B12.3 |  | **-** | **+** |  | Oliveira *et al.* [3] |
| Y39A1A.19 | *fmo-3* | **-** | **+** |  | Park *et al.* [4] |
| Y53F4B.32 | *gst-29* | **-** | **+** |  | Park *et al.* [4] |
| D1086.3 |  | **-** | **-** |  | Oliveira *et al.* [3] |
| F42A8.1 |  | **-** | **-** |  | Park *et al.* [4] |
| Y16B4A.2 |  | **-** | **-** |  | Oliveira *et al.* [3] |
| C32H11.12 | *dod-24* | **+** |  | **+** | Oliveira *et al.* [3] |
| F01D5.3 |  | **+** |  | **+** | Oliveira *et al.* [3] |
| F08G5.6 |  | **+** |  | **+** | Oliveira *et al.* [3] |
| F35E12.5 |  | **+** |  | **+** | Oliveira *et al.* [3] |
| K10C2.3 |  | **+** |  | **+** | Oliveira *et al.* [3] |
| T01D3.6 |  | **+** |  | **+** | Oliveira *et al.* [3] |
| Y40D12A.2 |  | **+** |  | **+** | Oliveira *et al.* [3] |
| F55G11.4 |  |  | **+** | **+** | Oliveira *et al.* [3] |
| C16H3.2 | *lec-9* | **+** |  |  | Oliveira *et al.* [3] |
| C26B9.5 |  | **+** |  |  | Oliveira *et al.* [3] |
| D1053.1 | *gst-42* | **+** |  |  | Oliveira *et al.* [3] |
| F52F10.4 | *oac-32* | **+** |  |  | Oliveira *et al.* [3] |
| F53E10.4 | *irg-3* | **+** |  |  | Oliveira *et al.* [3] |
| F56F10.1 |  | **+** |  |  | Oliveira *et al.* [3] |
| K10B2.2 |  | **+** |  |  | Oliveira *et al.* [3] |
| K12H4.7 |  | **+** |  |  | Oliveira *et al.* [3] |
| C05E11.4 | *amt-1* | **-** |  |  | Oliveira *et al.* [3] |
| C08F11.8 | *ugt-22* | **-** |  |  | Oliveira *et al.* [3] |
| F55E10.6 |  | **-** |  |  | Oliveira *et al.* [3] |
| F58B3.3 | *lys-6* | **-** |  |  | Oliveira *et al.* [3] |
| F58G6.3 |  | **-** |  |  | Oliveira *et al.* [3] |
| C12C8.2 | *cbl-1* |  | **+** |  | Oliveira *et al.* [3] |
| F25B4.8 |  |  | **+** |  | Oliveira *et al.* [3] |
| Y45G12C.2 | *gst-10* |  | **+** |  | Oliveira *et al.* [3], Park *et al.* [4] |
| F10D2.9 | *fat-7* |  |  | **+** | Oliveira *et al.* [3] |
| ZK666.6 | *clec-60* |  |  | **-** | Oliveira *et al.* [3] |
| B0218.6 | *clec-51* |  |  |  | Oliveira *et al.* [3] |
| C49C3.4 |  |  |  |  | Oliveira *et al.* [3] |
| F25D1.5 |  |  |  |  | Oliveira *et al.* [3] |
| K09C4.5 |  |  |  |  | Oliveira *et al.* [3] |
| K10C2.1 |  |  |  |  | Oliveira *et al.* [3] |
| T28F3.8 |  |  |  |  | Oliveira *et al.* [3] |
| Y105C5B.15 |  |  |  |  | Oliveira *et al.* [3] |
| Y34F4.1 |  |  |  |  | Oliveira *et al.* [3] |
| Y51H4A.24 |  |  |  |  | Oliveira *et al.* [3] |
| ZK455.4 | *asm-2* |  |  |  | Oliveira *et al.* [3] |

^#^Genes with >10 fold decrease in N2 15d adults compared to N2 6d adults [1].

*Characteristics of the effect: ‘+’ refers to genes up-regulated by PA14 infection, oxidative stress or PMK-1, ‘-‘ refers to genes down-regulated by PA14 infection, oxidative stress or PMK-1. Data was obtained from Wormbase [2].

Underlined sequences encode immune-related CUB-like proteins implicated in the antimicrobial response.

^$^Source of microarray data: genes whose expression changed by *skn-1(RNAi)* under non-stresse conditions from Oliveira *et al*. [3]; genes with SKN-1 dependent expression changes upon oxidative stress from Park *et al.* [4].

1. Youngman MJ, Rogers ZN, Kim DH (2011) A decline in p38 MAPK signaling underlies immunosenescence in *Caenorhabditis elegans*. PLoS Genet 7: e1002082.

2. Yook K, Harris TW, Bieri T, Cabunoc A, Chan J, et al. (2012) WormBase 2012: more genomes, more data, new website. Nucleic Acids Res 40: D735-741.

3. Oliveira RP, Porter Abate J, Dilks K, Landis J, Ashraf J, et al. (2009) Condition-adapted stress and longevity gene regulation by *Caenorhabditis elegans* SKN-1/Nrf. Aging Cell 8: 524-541.

4. Park SK, Tedesco PM, Johnson TE (2009) Oxidative stress and longevity in *Caenorhabditis elegans* as mediated by SKN-1. Aging Cell 8: 258-269.
