# Supplementary material for: A Role for SKN-1/Nrf in Pathogen Resistance and Immunosenescence in Caenorhabditis elegans
Source: PLoS Pathog. 2012 Apr 26;8(4):e1002673. doi: 10.1371/journal.ppat.1002673 (PMC3343120; doi:10.1371/journal.ppat.1002673)
Supplement: Table S3 — Statistical analysis of lifespan assays. (DOC) [file ppat.1002673.s007.doc]

**Table S3: Statistical analysis of lifespan assays**

**Survival data of *skn-1(zu135)* mutants on OP50 at 25^o^C**

| **Figure** | **Bacteria, trial** | **Strains and conditions** | **Mean survival (days)** | **N (total)** | **N (dead)** | **% change *vs.* N2** | **p *vs.* N2**  **(log rank)** |
| --- | --- | --- | --- | --- | --- | --- | --- |
| S4 | OP50, 1 | N2 | 10.6 | 51 | 49 |  |  |
|  |  | *skn-1(zu135)* | 8.6 | 50 | 43 | -18.8 | 0.0048 |
|  | OP50, 2 | N2 | 12.1 | 101 | 95 |  |  |
|  |  | *skn-1(zu135)* | 9.0 | 100 | 82 | -25.4 | <0.0001 |
|  | OP50, 3 | N2 | 14.1 | 100 | 94 |  |  |
|  |  | *skn-1(zu135)* | 12.1 | 50 | 48 | -14.1 | 0.0370 |

N (total) (total number of observations) = N (dead) + N (censored).
